# Partial Oxidation of Bio-methane over Nickel Supported on MgO–ZrO_2_ Solid Solutions

**DOI:** 10.1007/s11244-023-01822-7

**Published:** 2023-05-24

**Authors:** Yvan J. O. Asencios, Nevzat Yigit, Thomas Wicht, Michael Stöger-Pollach, Alessandra F. Lucrédio, Francielle C. F. Marcos, Elisabete M. Assaf, Günther Rupprechter

**Affiliations:** 1 Institute of Materials Chemistry, Technische Universität Wien, Getreidemarkt 9/BC/01, 1060 Vienna, Austria; 2 Institute of Marine Sciences, Universidade Federal de São Paulo, R. Maria Máximo 168, Santos, SP 11030-100 Brazil; 3 University Service Center for Transmission Electron Microscopy, Technische Universität Wien, Austria, Wiedner Hauptstraße 8-10, 1040 Vienna, Austria; 4 São Carlos Institute of Chemistry, Universidade de São Paulo, Av. Trab. São Carlense 400, São Carlos, SP 13566-590 Brazil

**Keywords:** Nickel catalyst, MgO–ZrO_2_ solid-solution, NiO–MgO solid-solution, One-step polymerization-method, Partial Oxidation of Methane, Synthesis gas

## Abstract

Syngas can be produced from biomethane via Partial Oxidation of Methane (POM), being an attractive route since it is ecofriendly and sustainable. In this work, catalysts of Ni supported on MgO–ZrO_2_ solid solutions, prepared by a one-step polymerization method, were characterized by HRTEM/EDX, XRD, XPS, H_2_-TPR, and in situ XRD. All catalysts, including Ni/ZrO_2_ and Ni/MgO as reference, were tested for POM (CH_4_:O_2_ molar ratio 2, 750 ºC, 1 atm). NiO/MgO/ZrO_2_ contained two solid-solutions, MgO–ZrO_2_ and NiO-MgO, as revealed by XRD and XPS. Ni (30 wt%) supported on MgO–ZrO_2_ solid solution exhibited high methane conversion and hydrogen selectivity. However, depending on the MgO amount (0, 4, 20, 40, 100 molar percent) major differences in NiO reducibility, growth of Ni^0^ crystallite size during H_2_ reduction and POM, and in carbon deposition rates were observed. Interestingly, catalysts with lower MgO content achieved the highest CH_4_ conversion (~ 95%), high selectivity to H_2_ (1.7) and CO (0.8), and low carbon deposition rates (0.024 g _carbon_.g_cat_^−1^ h^−1^) with Ni4MgZr (4 mol% MgO) turning out to be the best catalyst. In situ XRD during POM indicated metallic Ni nanoparticles (average crystallite size of 31 nm), supported by MgO–ZrO_2_ solid solution, with small amounts of NiO–MgO being present as well. The presence of MgO also influenced the morphology of the carbon deposits, leading to filaments instead of amorphous carbon. A combustion-reforming mechanism is suggested and using a MgO–ZrO_2_ solid solution support strongly improves catalytic performance, which is attributed to effective O_2_, CO_2_ and H_2_O activation at the Ni/MgO–ZrO_2_ interface.

## Introduction

A key concept in circular economy is to recycle and reuse. The valorization of biomass waste (e.g. agricultural waste) to produce clean energy thus enables a successful transition from the current linear to a circular economy of societies. The anaerobic digestion of biomass produces mainly biomethane and carbon dioxide, with minor proportion of other gaseous products such as H_2_S and NH_3_. The solid–liquid residue (digestate) is rich in nutrients and can be used as organic fertilizer. [1, 2]

Biomethane can play an important role in a circular economy, especially when further converted to Synthesis gas (Syngas, H_2_/CO). Syngas is crucial in industrial catalysis for liquid fuel production via Fischer–Tropsch (FT) synthesis, yielding clean fuels (free from S, N, etc.), which then emit less harmful pollutants. When further processed by water gas shift, syngas also represents a source of hydrogen, a promising energy vector for, e.g., fuel-cells. In general, fossil-free resources, more efficient processes and alternative routes are needed to reduce greenhouse gases, global warming and to meet international environmental agreements till 2030 [Paris Agreement; ONU, UNFCCC].

Currently, Syngas is mainly produced from methane (natural gas) via the steam reforming of methane (SRM), yielding a H_2_:CO ratio of 3:1. Alternatively, dry reforming of methane (DRM) utilizing CO_2_ yields H_2_:CO of 1:1. The main drawback is that both routes are based on fossil fuels and energy-demanding (endothermic), but these obstacles can be overcome.

First, methane can originate from biogas, also known as biomethane (purified methane from biogas), making it ecofriendly and sustainable. Second, the Partial Oxidation of Methane (POM, reaction 1) produces Syngas with less energy demand, because POM is exothermic, fast, and its H_2_/CO ratio is 2 (suitable for FT synthesis or methanol production).

1 $${\text{POM}}: \quad {\text{2 CH}}_{{4}} + {\text{ O}}_{{2}} \to {\text{ 4 H}}_{{2}} + {\text{ 2 CO}} \quad \Delta {\text{H}} = \, - {22}.{\text{6 kJ}}\,{\text{mol}}^{{ - {1}}}$$

Group VIIIB elements are good catalysts for methane reforming, especially the low-cost non-noble metals (for example Ni, Co, Fe). Industry uses Nickel-based catalysts for SRM, but they are suffering from carbon deposition (coking), which can lead to catalyst deactivation, and/or to an increase of reactor pressure (highly dangerous clogging [3–7]). Different ways have been suggested to minimize carbon deposition, including using solid solutions as catalyst supports (mixed oxides [3]), decreasing the crystallite size of the metal particles [8, 9], using metal alloys [10, 11], adding promoters, etc.

Herein, we used a combined strategy: (i) applying the POM reaction (O_2_ effectively removes carbon) and (ii) employing Nickel nanoparticles supported on MgO–ZrO_2_. Nickel nanoparticles supported on solid solutions (denoted “MgO–ZrO_2_”) work very well for POM, since the support is rich in oxygen vacancies important for activating oxygen, while avoiding total oxidation of CH_4_ to CO_2_. Solid solutions based on zirconia also have high oxygen storage capacity and excellent redox properties, which is beneficial for oxidation reactions, especially for POM. According to some reports [12, 13], these oxygen vacancies also aid in coke removal.

The effectivity of Ni/MgO–ZrO_2_ catalysts in POM was initially reported by Barbero et al. [14]. Later, Sun et al. [15] studied Ni/MgO–ZrO_2_ catalysts (prepared by coprecipitation) in the tri-reforming of coal bed methane to syngas, but the composition of the catalyst was not determined in detail [15]. It was argued that intense metal-support interaction, good thermal stability, and the basic nature of the catalyst were responsible for the good performance. Other work reported by Al-Fatesh et al. [16] studied the effect of MgO in NiO–ZrO_2_ catalysts (obtained by wet impregnation) in DRM. According to them, the interaction of NiO–MgO solid solution with the ZrO_2_ support was crucial and the reason for high CH_4_ and CO_2_ conversions. However, these authors [16] did not observe a stabilization of tetragonal zirconia (that is related to the formation of MgO–ZrO_2_), so that the role of oxygen vacancies during reaction was unknown. Favorable properties of Ni/MgO–ZrO_2_ catalysts were also reported by Titus et al. ([17], prepared by melt impregnation) for DRM and by Farooqi et al. ([18], co-precipitation/impregnation), applied for bi-reforming of methane. In all these studies, the exact origins of the favorable effects of MgO–ZrO_2_ (generating oxygen vacancies) and of NiO–MgO on catalytic performance and carbon removal remained rather unclear.

The current Ni/MgO–ZrO_2_ catalysts (prepared by a one-step polymerization method) were previously studied (with Ni content of 20 wt% in total) [12, 13, 19, 20] and it was demonstrated that two solid solutions were present: NiO–MgO and MgO–ZrO_2_. Both solid solutions influenced the catalytic behavior of the catalysts when used for oxidative reforming of methane (ORM) and POM, but their exact role was not understood. The major objective of the current study was thus to thoroughly characterize Ni/MgO–ZrO_2_ catalysts (with Ni at total weight content of 30%), including in situ techniques, to test their catalytic POM activity, and to elucidate the influence of the two solid solutions, NiO–MgO and MgO–ZrO_2_ on the POM reaction and the carbon deposition rates. The higher nickel content was chosen to aim for coke deposition and to examine the coke removal performance of the solid solutions under POM conditions.

## Experimental

### Preparation of Catalysts

The Ni-based catalysts were prepared by a One-Step Polymerization (OSP) method, previously reported in [12, 19], using Ni(NO_3_)_2_.6H_2_O, Zr(CO_3_)_2_.1.5H_2_O, Mg(NO_3_)_3._6H_2_O, citric-acid, and ethylene–glycol (all of analytical degree). This straightforward preparation method was chosen because it yields a very high dispersion of the catalysts´ components and a homogeneous texture. The as-synthesized polymers were subjected to calcination under an air stream in two consecutive steps at 500 and 750 °C, for a total of 5 h.

The molar percent of MgO in the support was varied: 0, 4, 20, 40, and 100%, of the total mol of the MgO–ZrO_2_ support: 0% means that the catalytic support is pure ZrO_2_, whereas for 100% the catalytic support is pure MgO. The amount of Ni was constant at 30% regarding the total weight of the catalyst (such a high loading is frequent for Ni reforming catalysts). Overall, this yielded 5 different catalysts under study labeled as NiZr, Ni4MgZr, Ni20MgZr, Ni40MgZr, and NiMg.

### Characterization Methods

#### X-ray Diffraction (XRD)

The crystal phases were identified by X-ray diffraction, in a Rigaku Multiflex X-ray diffractometer (40 kV, 30 mA), each analysis scanned in the range 2θ = 5°–80° (at 2° min^−1^), using Cu Kα radiation as source (λ = 1.5406 Å). The crystal phases were identified by matching the available data in the International Center of Diffraction Data (ICDD-JCPDS).

#### In Situ XRD

The in situ XRD analysis was carried out on a PANalytical X'Pert Pro diffractometer in Bragg–Brentano geometry using Cu Kα1,2 radiation filtered with an BBHD mirror and an X’Celerator linear detector, the wavelength used for each analysis was 1.5406 Å. Each analysis recorded a 2θ region of 35°–45° (containing the principal peaks of Ni^0^ and NiO). In every test, approximately 100 mg of the powder catalyst was placed in a ceramic sample holder in a temperature-programmable oven. For in situ experiments, an Anton Paar 900 high temperature chamber was used with the sample temperature monitored/controlled via a thermocouple and heating. Each catalyst was in contact with the respective gas flow. The first XRD pattern was acquired at room temperature in a flow of Ar (100 mL·min^−1^). The catalyst was then heated from room temperature to 750 °C in 30 mL·min^−1^ H_2_ stream (approx. 5% H_2_/He), at a rate of 10 °C·min^−1^. Subsequent XRD patterns were collected in situ at 50, 150, 250, 350, 450, 550, 650, and 750 °C. After this procedure, the cell was purged with Ar. Another XRD pattern was acquired in situ after 1 h of POM reaction, exposing the reduced catalyst to a mixture of CH_4_ (33 mL·min^−1^), O_2_ (16.5 mL·min^−1^) and Ar (60 mL·min^−1^) at 750 °C.

The average crystallite sizes (*d*) of the catalysts were determined from XRD and in situ XRD using the Scherrer Equation (*d* = k·λ/(β_hkl_·cosθ)), where k is the shape factor (= 0.89), λ is the wavelength of CuKα radiation, θ is Bragg’s angle and β_hkl_ is the full width at half maximum (FWHM) of the principal peak of the crystal phase.

#### Temperature Programmed Reduction (TPR)

Analysis by Temperature Programmed Reduction with H_2_ (H_2_-TPR) was carried out in a multi-purpose quartz reactor, with the H_2_ consumption measured (in-line) with a Thermal Conductivity Detector (TCD). The quantification of H_2_ consumption of each catalyst was calculated by comparing the corresponding peak area to that of a standard CuO powder. For each TPR analysis, 100 mg of catalyst and a gaseous mixture containing hydrogen (1.96% H_2_/Ar, flowing at 30 mL·min^−1^) were used. The analyses were carried out in the temperature range of 25–1000 °C (at a heating rate of 5 °C·min^−1^).

#### Electron Microscopy

High-resolution transmission electron microscopy (HRTEM), high-angle annular dark-field scanning transmission electron microscopy (HAADF-STEM), electron energy loss spectrometry (EELS) and energy filtered TEM (EFTEM) were performed using a 200 kV FEI Tecnai F20 S-TWIN analytical (scanning) transmission electron microscope [(S)TEM]. The catalysts were directly deposited on carbon-coated copper grids, and contamination and adsorbed water were removed by plasma cleaning*.*

#### Energy-Dispersive X-ray (EDX) Spectroscopy

The chemical composition of the catalysts was determined by energy-dispersive X-ray spectroscopy (EDX), in a LEO 440 scanning electron microscope (using a tungsten filament coupled to an EDX detector), with three different regions of each sample analyzed.

#### X-ray Photoelectron Spectroscopy (XPS)

All X-ray photoelectron spectroscopy (XPS) experiments were carried out at room temperature in a stainless-steel UHV chamber (35 l, base pressure < 5 × 10^–10^ mbar) for surface analysis, equipped with a Specs XR50© high intensity nonmonochromatic Al/Mg dual anode X-ray source and a Phoibos 100© hemispherical energy analyzer (EA) with multichannel plate detector. The conditions used were: Al anode at 1486.6 eV, with the value of 285.0 eV for C1s at 0° emission angle serving as a binding energy reference. XPS data were treated by analysis via CasaXPS software.

#### Specific Surface Area (SSA)

The SSA of each catalyst was measured in a Quantachrome Nova 1200 instrument, through N_2_ adsorption/desorption isotherms at liquid nitrogen temperature. The results were treated according to the Brunauer–Emmett–Teller (BET) method.

### Catalytic Tests

Catalytic tests were carried out in a fixed-bed down-flow quartz reactor (i.d. = 10 mm), and in every test 100 mg of catalyst was used. Before reactions, the catalysts were pre-treated with H_2_ (30 mL·min^−1^) at 750 °C for 30 min (to reduce NiO to Ni^0^). After H_2_ pre-treatment, the catalyst surface was cleaned with inert gas (N_2_) at the same temperature, followed by POM reaction for 5 h at 750 °C, with an inlet gas mixture with a molar ratio of 2 CH_4_:1 O_2_, composed of 32 mL·min^−1^ (CH_4_) and 16 mL·min^−1^ (O_2_), with oxygen added as synthetic air (60 mL·min^−1^ of nitrogen and 16 mL·min^−1^ of oxygen), thus the total flow in the reactor was 108 mL·min^−1^. The reactor was connected in-line to a gas chromatograph (Varian, Model 3800). A thermocouple inserted directly in the catalyst bed was used to measure and control the reaction temperature.

The gas chromatograph was equipped with an automated injection valve, two chromatographic columns and two thermal conductivity detectors (TCD): hydrogen and methane were separated on a 13X molecular sieve packed column, with nitrogen as the carrier gas, while N_2_, CO_2_, CH_4_, and CO were separated on a Porapak-N packed column, with He as the carrier. All catalytic tests were carried out twice and the values plotted in the respective figures are average values.

The carbon deposition rates (mmol·h^−1^) were calculated as the apparent mass gain of the catalysts after a specific POM reaction time.

The CH_4_ conversion was calculated as:

$${\text{Conversion CH}}_{{4}} \, = \, \left( {{\text{Mol CH}}_{{{4}\,{\text{in}}}} - {\text{ Mol CH}}_{{{4}\,{\text{out}}}} } \right)/\left( {{\text{Mol CH}}_{{{4}\,{\text{in}}}} } \right)$$

The H_2_, CO, CO_2_ selectivities were calculated as:

$${\text{Selectivity i }} = {\text{ Mol of i}}_{{{\text{produced}}}} {\text{/Mol of CH}}_{{{4}\,{\text{converted}}}}$$

where i = product (H_2_, CO or CO_2_).

### Post-reaction Characterization of the Used Catalysts

#### Carbon Deposition

The carbon deposition rates (mmol·h^−1^) were determined from the apparent mass gain of the catalysts after a specific POM reaction time, with respect to the reduced catalysts (H_2_ pre-treated at 750 °C for 30 min) before reaction.

#### Thermogravimetric Analysis (TGA)

The coke produced after the catalytic tests was characterized by thermogravimetric analysis (TGA), using a Shimadzu DTG-60H simultaneous TG/DTA analyzer, using a continuous air stream flow and a heating rate of 10 °C∙min^−1^.

#### Scanning Electron Microscopy (SEM)

The used catalysts were analyzed by Scanning Electron Microscopy (SEM), LEO model 440, equipped with an Oxford detector, operating at 20 kV. Each sample was coated with a gold layer to avoid charge build-up.

## Results and Discussion

### Characterization of Calcined Catalysts

#### TEM

**Fig. 1:**
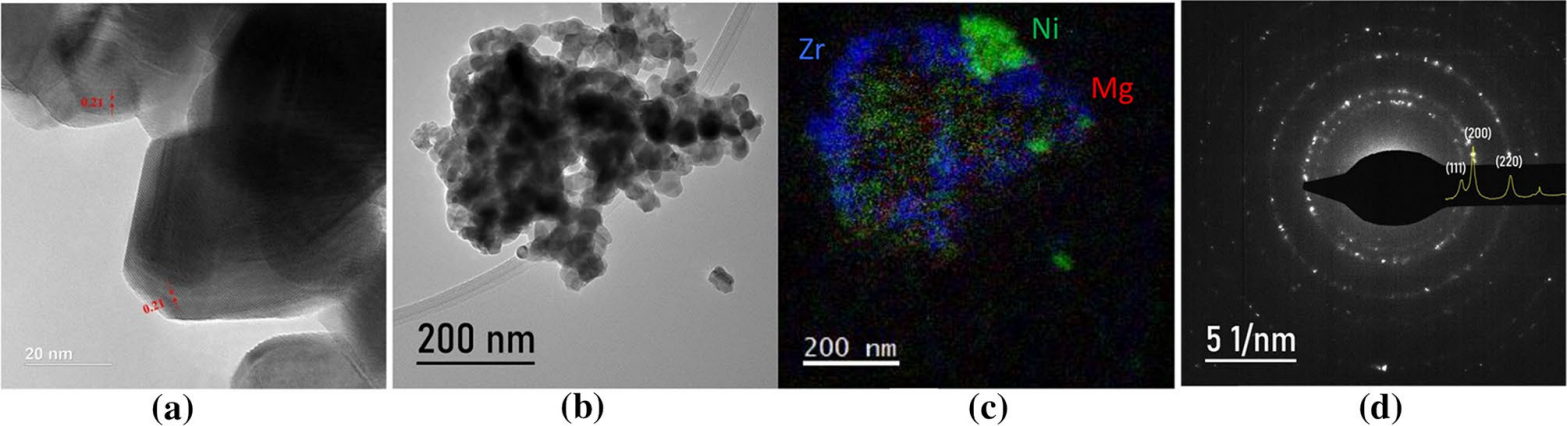
**a** HRTEM of NiZr; **b** overview and **c** EFTEM of Ni4MgZr; **d** SAED pattern of NiMg catalyst

To investigate the internal elemental distribution of catalyst particles, (S)TEM was applied in combination with EELS and EFTEM. Figure 1a–c display images of NiZr, Ni4MgZr, and NiMg, respectively. In Fig. 1a, a NiO particle with a dominant (100) facet is imaged in high resolution. The corresponding EELS spectrum (not shown) clearly revealed stoichiometric NiO, which was confirmed by EELS quantification and by comparing the energy loss near edge structure (ELNES) of the Ni-L edge with literature. The supporting ZrO_2_ had the shape of nano-spheres.

For Ni4MgZr, Fig. 1b shows an overview of a conglomerate of particles, which are chemically quantified in Fig. 1c by means of EFTEM. As can be seen in the diffraction pattern in Fig. 1d, the Ni_(x)_Mg_(1−x)_O catalyst shows the lattice parameters of MgO (0.412 nm).

#### X-ray Diffraction

**Fig. 2:**
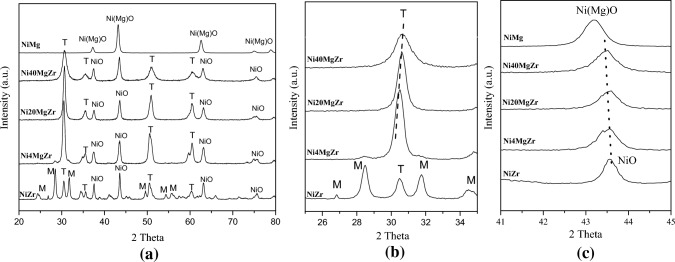
XRD patterns of the calcined catalysts: **a** complete XRD patterns, **b** enlargement of the XRD pattern for 2θ = 20°–35°; **c** enlargement for 2θ = 41°–45º. T: tetragonal phase of ZrO_2_, M: monoclinic phase of ZrO_2_, NiO: cubic crystal face (*fcc*) or cubic solid solution (*fcc*) NiO–MgO

Figure 2a collects the XRD patterns of the fresh catalysts (after calcination), with Fig. 2b and c showing enlarged regions of 25°–35º and 41º–45º, respectively. According to Fig. 2a, peaks assigned to the tetragonal phase of ZrO_2_ (JCPDS 17-923) are present in all zirconium-containing samples, while the monoclinic phase of ZrO_2_ is additionally observed only in NiZr. After addition of MgO (Ni4MgZr, Ni20MgZr, and Ni40MgZr), the monoclinic phase was no longer present, because the MgO–ZrO_2_ solid solution stabilized the tetragonal ZrO_2_ structure, as reported previously [13, 20]. In Fig. 2b, a continuous shift of the ZrO_2_ principal peak to larger Bragg angles is observed for higher MgO content, indicating that the ZrO_2_ crystal lattice was successively contracted upon formation of the MgO–ZrO_2_ solid solution. Lattice parameters calculated from the (111) peak of the tetragonal phase of ZrO_2_ are summarized in Table 1.

**Table 1:** Properties of calcined catalysts calculated from XRD

Catalysts	Lattice parameter “*a*” (Å)		Average crystallite size (nm)	
	ZrO_2_ tetragonal	NiO *fcc*	ZrO_2_	NiO
NiMg	n.a	2.093	n.a	15.8
Ni40MgZr	5.043	2.080	8.0	15.2
Ni20MgZr	5.058	2.079	14.2	17.1
Ni4MgZr	5.075	2.076	14.7	16.8
NiZr	5.078	2.075	20.1	25.2

The contraction can be explained by the ionic radius of Mg^2+^(0.57 Å) being smaller than that of Zr^4+^ (0.59 Å). Therefore, during catalyst preparation at high temperature the Mg^2+^ ions substituted some Zr^4+^ ions in the ZrO_2_ crystal lattice, forming a substitutional solid-solution [21]. The contraction increases as more MgO is added. The formation of the MgO–ZrO_2_ solid solution also involves the generation of oxygen vacancies to maintain electro-neutrality due of the lower oxidation state of the cations at some sites (Mg^+2^ vs. Zr^+4^). Furthermore, as the MgO quantity was increased, the diffraction peaks of tetragonal ZrO_2_ became broader.

MgO addition also caused a shift of NiO peaks to lower Bragg angles (Fig. 2c), pointing to the formation of a NiO–MgO solid solution, as noted in previous reports for similar conditions [13, 20]. The formation of the NiO–MgO solid solution leads to an enlargement of the lattice parameter of NiO (Table 1), coherent with the ionic radius of Mg^2+^ (0.57 Å) being larger than that of Ni^2+^ (0.55 Å [21]), suggesting that Mg^2+^ cations entered the cubic lattice of NiO forming a substitutional solid-solution. This is favored by the structural similarity of NiO and MgO, having face-centered-cubic structures, identical cation charges (Ni^2+^ and Mg^2+^), and similar bond distances (2.10 and 2.11 Å for Ni–O and Mg–O, respectively) [22].

A distinct main peak of cubic MgO located at 43.10° (JCPDS 78-0430), observed for NiMg, is absent in the XRD patterns of Ni4MgZr, Ni20MgZr, and Ni40MgZr. This indicates that MgO is taking part in both solid solutions (NiO–MgO and MgO–ZrO_2_), once more suggesting that Mg^2+^ cations replaced Zr^4+^ ions in the ZrO_2_ crystal lattice and Ni^2+^ ions in the NiO crystal lattice.

The crystallite sizes calculated from the Scherrer formula are included in Table 1. These values suggest that the addition of MgO to NiZr reduced the size of both tetragonal ZrO_2_ and cubic (*fcc*) NiO crystallites.

#### Specific Surface Area (BET)

Table 2 shows the specific surface area of each catalyst, obtained by N_2_ adsorption using the BET method, with higher values upon increasing MgO content. This agrees with the smaller crystallite sizes of ZrO_2_ (see Table 1) formed at higher MgO content.

**Table 2:** Specific surface area of the catalysts obtained by N_2_ adsorption using the BET method

Catalysts	Specific surface area (m^2^·g^−1^)
NiMg	31
Ni40gZr	24
Ni20MgZr	17
Ni4MgZr	15
NiZr	12

#### EDX

The elemental composition of each catalyst was determined by energy-dispersive X-ray spectroscopy (EDX), with results shown in Table 3. The obtained values are close to the nominal composition aimed for in the synthesis, i.e. 30%wt. Ni and molar % of Mg of 4, 20, and 40.

**Table 3:** Chemical composition of the catalysts, obtained by EDX

Catalysts	wt% (EDX)	Atomic % (EDX)				
	Ni	Ni	Mg	Zr	O	Mg/(Mg + Zr), %
NiMg	29.5	12.6	44.8	0.0	42.6	100.0
Ni40MgZr	32.3	20.3	10.3	15.1	54.2	40.6
Ni20MgZr	31.1	21.5	6.0	19.7	52.8	23.3
Ni4MgZr	30.3	22.5	1.4	23.7	52.3	5.6
NiZr	31.3	24.7	0.0	26.3	49.0	0.0

#### XPS

**Fig. 3:**
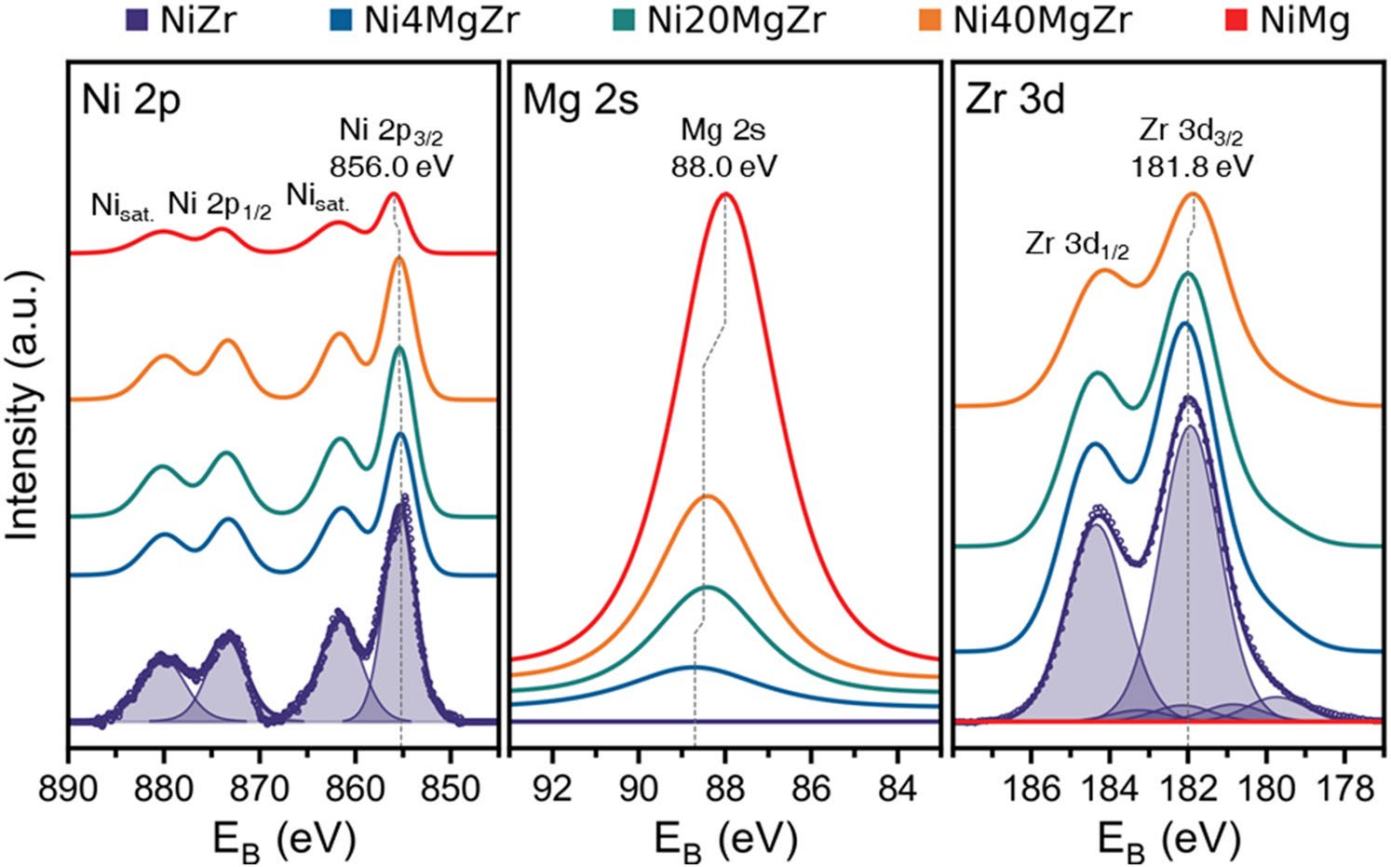
XPS Ni 2p, Mg 2s and Zr 3d spectra of the calcined catalysts

Complementing EDX, X-ray photoelectron spectroscopy (XPS) was applied to obtain information about the surface composition of the catalysts. Based on the used photon energy and the inelastic mean free paths (IMFPs) of Ni, Zr and Mg (13, 23, and 24 Å nm, respectively), the corresponding probing depth is up to 6 nm. Figure 3 displays the XPS Ni 2p, Mg 2 s, and Zr 3d, spectra, respectively, with Table 4 collecting the corresponding binding energies (E_B_, eV) and surface composition.

**Table 4:** Binding energy values (E_B_, eV) and atomic % obtained by XPS analysis

Catalysts	Ni (2p)	Zr (3d)	Mg (2s)	O (1s)^a^	Ni (2p)	Zr (3d)	Mg (2s)	O (1s)
	Binding energy (E_B_, eV)				Atomic percent XPS, %			
NiMg	856.0		88.0	529.9	2.2	–	49.0	48.8
Ni40MgZr	855.3	181.8	88.4	529.9	4.8	22.0	19.7	53.5
Ni20MgZr	855.1	182.0	88.4	529.9	5.6	26.6	10.7	57.1
Ni4MgZr	855.0	182.0	88.7	530.0	4.7	32.3	4.8	58.2
NiZr	854.9	182.0	–	530.0	7.7	31.2	–	61.1

^a^Binding energy of the highest intensity component corresponding to bulk oxide

The line shapes and energies of both the Ni 2p (Fig. 3) and Ni LMM region (not shown) agree with literature data of NiO (BE Ni 2p_3/2_: 855.4 eV (highest intensity peak); BE Ni LMM ~ 844 eV) [23, 24]. The increase of the Ni 2p3/2 BE from 854.9 to 856.0 eV upon increasing Mg content points to the formation of a NiO·MgO solid solution [25] or to larger NiO particle size [26, 27].

For NiMg (Fig. 3), the Mg 2s BE of 88.0 eV corresponds to bulk MgO (literature value of 87.8 eV ([28]). A BE shift up to − 0.7 eV upon increasing in content may again be connected to varying amounts of NiO·MgO solid solution.

Figure 3 also shows the Zr 3d core level spectra. For, NiZr, the Zr 3d_5/2_ binding energy of ~ 182 eV matches well both theoretical [29] and experimental ([30, 31]) data of ZrO_2_. A minor increase in BE for the Mg containing catalysts may arise due to the change in coordination number of Zr atoms: according to XRD, only tetragonal ZrO_2_ was present in these samples (in NiZr, ZrO_2_ is both tetragonal and monoclinic). Independent of Mg content, additional weak doublets of Zr were found for all samples at ~ 1 and ~ 2 eV lower binding energies, likely corresponding to ZrOx suboxides or thin ZrO_2_ layers [31].

Apart from the mere BEs, the XPS signal intensities are informative. A comparison of the sample surface vs. bulk composition measured by XPS (Table 4) and EDX (Table 3), respectively, reveals significant differences. The amount of Ni at the surface was 3–6 times smaller than in the bulk. Apparently, the abundance of Zr and Mg at the surface leads to smaller Ni signals.

Mg surface segregation was reported for air calcination of Ni–Mg alloy films ([32]), as well as for Ni/MgO, prepared by incipient wetness impregnation, finally leading to the formation of a NiO·MgO solid solution [25, 33]. This picture is further confirmed by the fact that the ratio of Ni(XPS)/Ni(EDX) decreases for increasing Mg amounts in the sample. The surprising observation that Ni(XPS)/Ni(EDX) is still ~ 0.3 for NiZr (with Mg absent) may be explained by incorporation of Ni^2+^ into ZrO_2_, partly forming a NiO·ZrO_2_ solid solution sufficient to stabilize the tetragonal ZrO_2_ phase [34, 35]

#### H_2_-TPR

**Fig. 4:**
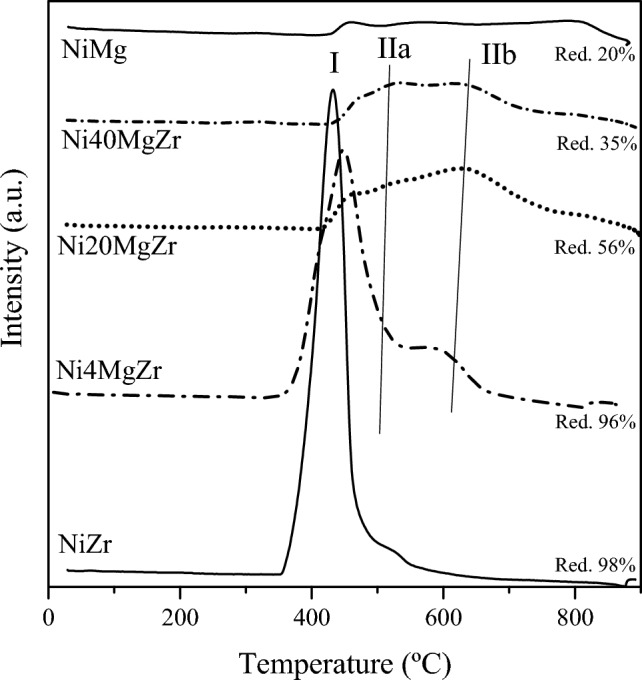
TPR profiles of catalysts (Red. = reduction %)

Following the characterization of calcined samples, TPR was applied to examine the reducibility of oxide species in the catalysts. Figure 4 displays TPR profiles of all samples and the extent of reduction (%) of each catalyst. The reduction of ZrO_2_ and MgO–ZrO_2_ supports up to 1000 °C is almost insignificant, as described in [12, 13]. Therefore, the TPR profiles are attributed to the reduction of NiO (NiO + H_2_ → Ni^0^ + H_2_O). Three peaks can be distinguished at low (peak I) and high temperatures (peaks IIa and IIb), related to NiO species weakly and strongly interacting with the support, respectively. According to TPR, already the addition of 4% MgO to NiO/ZrO_2_ favored the formation of NiO species strongly interacting with the support (peak IIa, located at higher temperatures than peak I). Further addition of MgO (Ni20MgZr and Ni40MgZr) led to even more strongly bound NiO species (peak IIb). As with increasing MgO content all peaks shifted to higher temperatures, continuous formation of a NiO–MgO solid solution is suggested. The oxygen vacancies produced in the MgO–ZrO_2_ solid solution (as previously reported in [13, 20]]), apparently do not favor NiO reduction. In NiMg, a well-estsablished NiO–MgO solid solution nearly fully inhibits NiO reduction, owing to electron transfer from NiO to MgO.

#### In Situ XRD Upon H_2_ Reduction

**Fig. 5:**
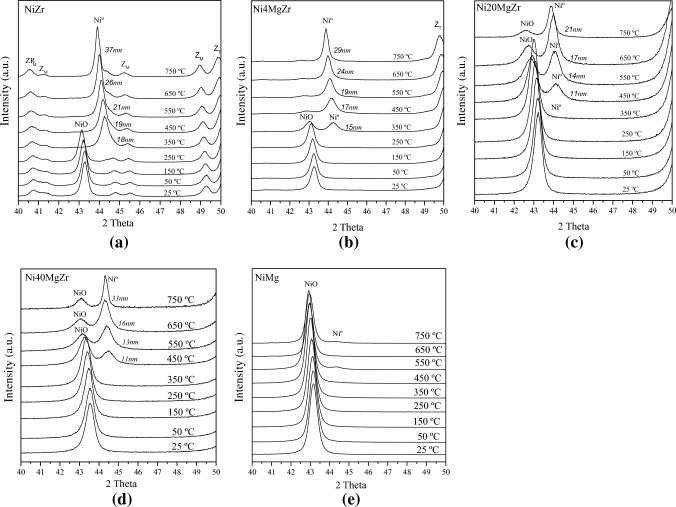
XRD patterns of catalysts during reduction in H_2_ (from 25 to 750 ºC). **a** NiZr, **b** Ni4MgZr, **c** Ni20MgZr, **d** Ni40MgZr, **e** NiMg

In this type of analysis, the catalysts were characterized by in situ XRD during reduction in H_2_ (Fig. 5), analogous to TPR. Figure 5a corresponds to the NiZr catalyst, indicating that the reduction of NiO to Ni^0^ set in below 350 ºC, in agreement with TPR (Fig. 4). The peak shift to lower Bragg angles upon further heating is related to the expansion of the crystal lattice at high temperature. During H_2_ reduction, the crystallite size of Ni^0^ in NiZr increased from 18 to 37 nm.

Figure 5b–d show the in situ XRD analysis during H_2_ reduction of Ni4MgZr, Ni20MgZr, and Ni40MgZr, indicating an onset of NiO reduction around 350, 450, and 450 ºC, respectively. The mean crystallite size of Ni^0^ increased from 15 to 29 nm, 11 to 21 nm, and 11 to 33 nm, respectively. Accordingly, the reached Ni particle size was smaller for MgO-containing catalysts, which is beneficial as smaller Ni^0^ crystallites are known to favor catalysis and limit carbon deposits. It seems that MgO–ZrO_2_ solid solutions hinder the crystal growth of Ni^0^ during reduction, especially for 4 and 20% MgO. For Ni40MgZr, even at 750 ºC NiO was still present, as in this catalyst a larger quantity of NiO–MgO solid solution is formed. The latter can be understood by the in situ XRD of reduction of NiMg (Fig. 5e), with mostly NiO and only a tiny amount of Ni^0^ present even at 750 ºC. As expected, the NiO–MgO solid solution cannot be reduced under these conditions.

### Catalytic Tests

#### Conversion and In Situ XRD

**Fig. 6:**
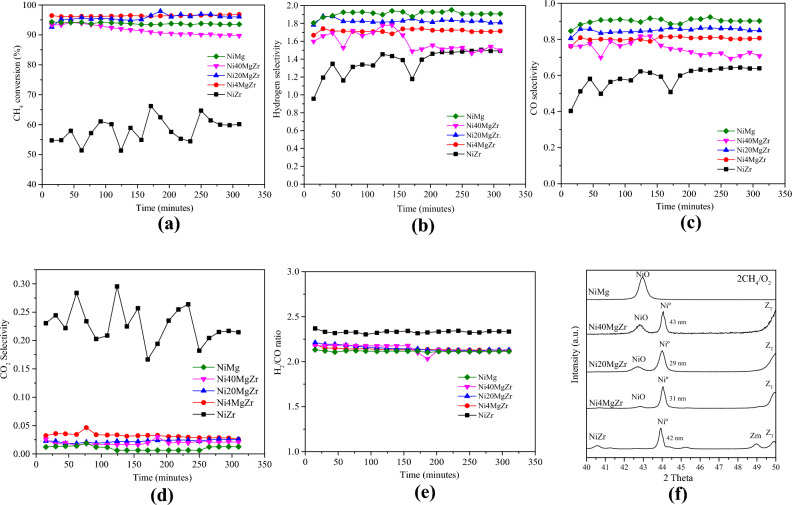
Catalytic test for POM. **a** Conversion of methane for various catalysts. **b** Selectivity to H_2_. **c** Selectivity to CO. **d** Selectivity to CO_2_. **e** H_2_/CO ratio. **f** in situ XRD collected at 750 °C, after 1 h of POM

The different pre-reduced catalysts were then tested for POM with results summarized in Fig. 6a–e. The average conversions of methane over Ni4MgZr, Ni20MgZr, Ni40MgZr, and NiMg, shown in Fig. 6a, are in a range of 93–96%, much higher than for NiZr (around 60%). However, for NiZr, the pronounced growth of Ni^0^ crystallites during reduction (Fig. 5a) and during POM (cf. Figure 6f) can partly explain its lower conversion.

Ni4MgZr and Ni20MgZr exhibited the highest catalytic conversion (Fig. 6a), and according to in situ XRD (Figs. 5b,c, and 6f), they also maintained smaller Ni^0^ crystallite size during reduction and during POM. The methane conversion over Ni40MgZr decreased with reaction time, probably because on the higher amount of MgO more carbonates formed that poisoned the active centers [36]. Ni sintering (Fig. 6f) may also contribute to the decreasing activity. For NiMg, mostly NiO is present during POM, but despite its minute Ni^0^ surface concentration, the conversion was still quite high. As reported by [22, 37–39], the NiO–MgO solid solution provides a good Ni distribution in the catalyst.

For all samples, the initial Ni^0^ crystallite size of the reduced catalysts increased within 1 h of POM reaction (Figs. 5a–d and 6f): NiZr (from 37 to 42 nm), Ni4MgZr (from 29 to 31 nm), Ni20MgZr (from 21 to 29 nm), and Ni40MgZr (from 33 to 43 nm).

#### Selectivity and Mechanism

Figure 6b and c display the selectivities to H_2_ and CO, respectively, monitored over reaction time. The selectivities are high and quite similar for Ni4MgZr, Ni20MgZr and NiMg, but lower for Ni40MgZr and much lower for NiZr.

Two general mechanisms have been proposed for Syngas production by POM: (i) a combustion-reforming mechanism, which initially involves the fast total combustion of methane (TCM), followed by Dry Reforming of Methane (DRM) and Steam Reforming of Methane (SRM), as shown in reactions (2–4); (ii) a pyrolysis mechanism, in which Syngas is produced directly according to reaction (1). Both mechanisms lead to H_2_ and CO at a molar ratio of 2 (in case of (i), the global sum TCM + DRM + 2 × SRM yields POM).

2 $${\text{TCM}}: {\text{CH}}_{{4}} + {\text{ 2O}}_{{2}} \to {\text{ CO}}_{{2}} + {\text{ 2H}}_{{2}} {\text{O}} \quad \Delta {\text{H}} = - {89}0{\text{ kJ}}\,{\text{mol}}^{{ - {1}}}$$

3 $${\text{DRM}}: {\text{CH}}_{{4}} + {\text{ CO}}_{{2}} \to {\text{ 2CO}} + {\text{ 2H}}_{{2}} \quad \Delta {\text{H}} = { 26}0.{\text{5 kJ}}\,{\text{mol}}^{{ - {1}}}$$

4 $${\text{SRM}}: {\text{CH}}_{{4}} + {\text{ H}}_{{2}} {\text{O}} \to {\text{CO}} + {\text{ 3H}}_{{2}} \quad \Delta {\text{H}} = { 225}.{\text{4 kJ}}\,{\text{mol}}^{{ - {1}}}$$

In the catalytic tests, traces of water and CO_2_ were indeed detected, which is why the combustion-reforming mechanism is favored for the studied catalysts.

Figure 6d shows the CO_2_ selectivity during POM. Almost all catalysts have very low selectivity to CO_2_, only for NiZr the values are higher. Apparently, POM over NiZr involves TCM. The inherent oxygen vacancies present in ZrO_2_ (tetragonal and monoclinic) are active in oxygen activation increasing the number of O_(s)_ species [40–42], responsible for TCM and the resulting CO_2_.

The MgO–ZrO_2_ solid solution, and smaller amounts of NiO–MgO, formed in Ni4MgZr, Ni20MgZr and Ni40MgZr, thus favored the activation of CO_2_ and H_2_O. As reported in [43, 44] their oxygen vacancies dissociate CO_2_, resulting in a release of CO and chemisorbed O_(s)_ species (CO_2_ + oxygen vacancy → CO + O_(s)_). O_(s)_ can then react with CH_4_. Similarly, the oxygen vacancies interact with H_2_O forming H_2_ and chemisorbed O_(s)_ species [43]. This is consistent with previous reports [45–48] on the beneficial effects of zirconia based solid-solutions in DRM and SRM.

All observations point to an important catalytic role of the interface sites between Ni^0^ and MgO–ZrO_2_, but also of NiO–MgO, with the latter increasing for higher MgO content. The oxygen vacancies present in Ni4MgZr, Ni20MgZr, and Ni40MgZr are already beneficial for POM, but it seems that also the basic centers in these catalysts (owing to the basic character of MgO) may enhance CO_2_ and H_2_O activation, i.e. DRM (Reaction 3) and SRM (Reaction 4). Consequently, the selectivity to CO is increased (cf. Figure 6c). The effect of basic centers is apparent from the results of NiMg (highest selectivity to H_2_ and CO). The role of MgO is to form intermediate carbonate species that rapidly decompose, enabling the further conversion of CO_2_ formed during POM [49, 50]. In summary, it is suggested that NiMgZr catalysts benefit both from active interfaces/oxygen vacancies (owing to MgO–ZrO_2_ and to a lesser extent NiO–MgO) and basic centers (owing to MgO).

#### H_2_/CO Ratio

Based on the results so far, POM likely occurs via a combustion-reforming mechanism. Nevertheless, on NiZr TCM is still significant, whereas the other catalysts favor subsequent DRM and SRM. This trend is also reflected by the H_2_/CO ratio (Fig. 6e): as NiZr produced more CO_2_ than CO, a higher H_2_/CO ratio (> > 2) is obtained. On the other hand, for the other catalysts (Ni4MgZr, Ni20MgZr, Ni40MgZr, and NiMg) the H_2_/CO ratio was very close to 2 (which is the stoichiometric ratio of Syngas produced by POM 2CH_4_:1O_2_). Once more, this suggests that these catalysts promote DRM and SRM with similar contributions, so that the H_2_/CO ratio is closer to the stoichiometric value. The role of TCM, DRM, and SRM in POM over different catalysts was also described in [51]**.**

### Post Reaction Analysis

#### Carbon Deposition Rates

Table 5 collects the (absolute) carbon deposition rate of each catalyst, measured after 5 h of POM, yielding an order of: Ni20MgZr≈Ni40MgZr > NiMg >  > Ni4MgZr > NiZr. Please note that during reaction Ni is mostly metallic (Fig. 6f), which is why the reduced catalysts serve as starting point (the partial oxidation to NiO upon reaction is insignificant). Table 5 also shows the average values of conversion of methane, and selectivity for H_2_ and CO. Table 5 also compares the carbon deposition rate normalized by the average conversion and relative to NiZr. The best catalyst is thus Ni4MgZr, as it had the highest methane conversion (Fig. 6a), good selectivity to H_2_ and CO, but still a low carbon deposition rate. It was previously suggested [52, 53] that large nickel particles favor carbon deposition, so that the larger increase in Ni^0^ crystallite size of Ni20MgZr and Ni40MgZr may contribute to their higher carbon deposition rates (Table 4). The lowest carbon deposition rate observed for NiZr is not a good comparison, as it also had much lower conversion.

**Table 5:** Carbon deposition rates (based on weighthing the reduced catalysts before reaction and the used catalysts after reaction)

Catalysts	g _carbon_ g_cat_^−1^ h^−1^	CH_4_ conversion (average values, %)	Selectivity to H_2_ (average, %)	Selectivity to CO (average, %)	Carbon deposition carbon rate normalized by conversion
NiMg	0.094	93.8	1.9	0.9	9.4/0.938≈10
Ni40MgZr	0.118	91.9	1.6	0.7	11.8/0.919≈12.8
Ni20MgZr	0.126	95.9	1.8	0.8	12.6/0.959≈13.1
Ni4MgZr	0.024	96.4	1.7	0.8	2.4/0.964≈2.5
NiZr	0.006	58.2	1.4	0.6	0.6/0.582≈1

Oxygen vacancies (e.g. of the MgO–ZrO_2_ solid solution) favor oxygen dissociation to form various reactive oxygen species adsorbed on the surface of the catalyst: O_2_^−^, O_2_^2−^, O^2−^, O^−^ [40, 42], which may remove carbon deposits by reacting with the surface carbon species (CH_x_ + O_(s)_ → CO + x H; x = 1,2,3). However, it appears that a higher MgO amount in the NiO–MgO–ZrO_2_ mixture was not beneficial for lowering carbon deposits during POM. Although NiMg also had high methane conversion, its carbon deposition rate was quite high, which may be due to the absent MgO-ZrO_2_ solid solution.

It seems that the excess of MgO in the NiO–MgO–ZrO_2_ mixture was not beneficial for reducing carbon deposits during the POM reaction, probably because the excess of MgO formed more carbonates that affected the oxygen vacancies required for carbon removal. A previous study [36] demonstrated that this excess also hindered methane decomposition in the course of DRM. Among the catalysts studied herein, a composition of Ni4MgZr was best for limiting carbon deposits.

#### SEM

**Fig. 7:**
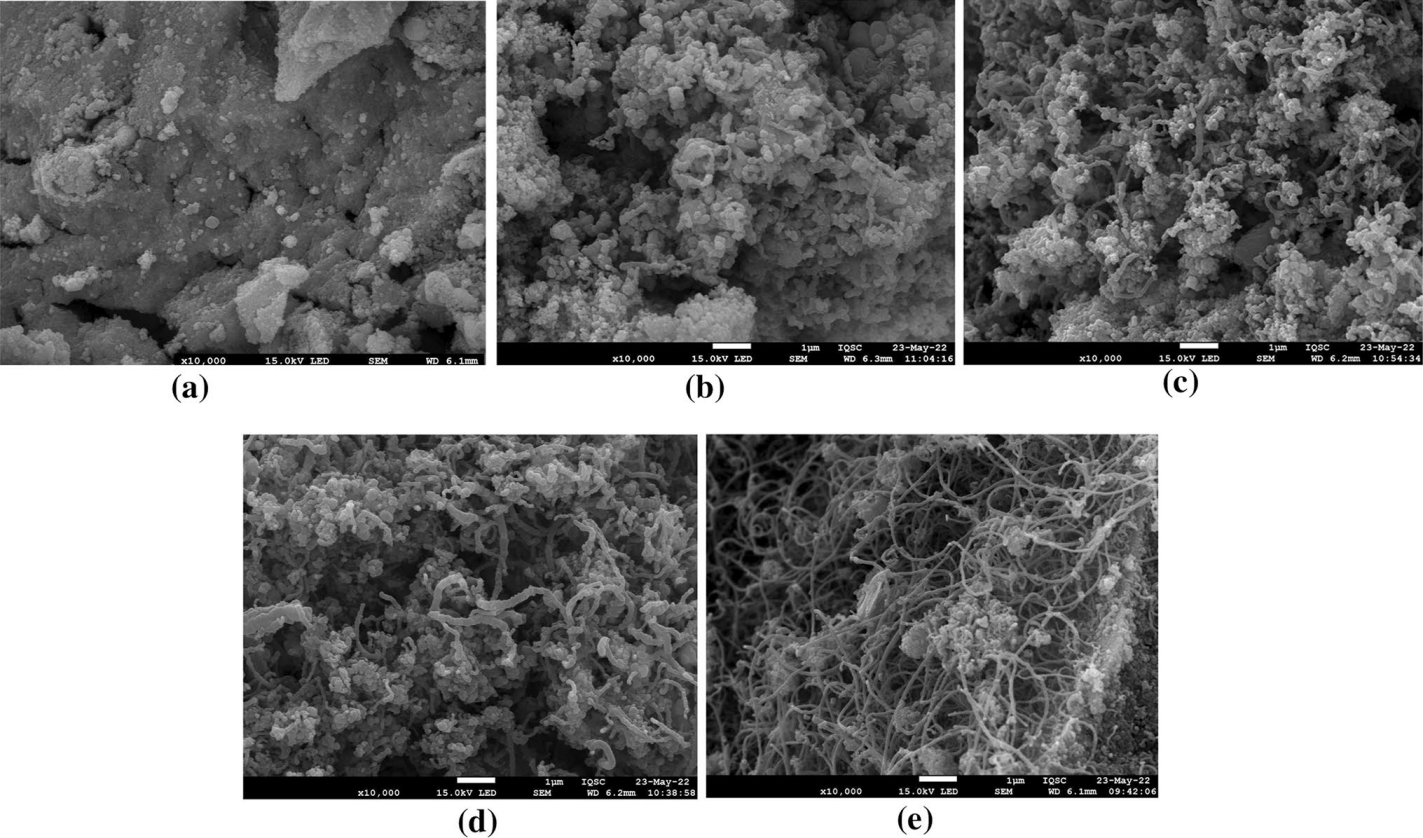
**a–e** Scanning Electron Microscopy (SEM) images of used catalysts: **a** NiZr; **b** Ni4MgZr; **c** Ni20MgZr; **d** Ni40MgZr; **e** NiMg

The coke formed during POM and deposited on each catalyst was analyzed by SEM, with Fig. 7a–e showing the morphology of the used catalysts. On NiZr, amorphous carbon was formed, as no defined shapes were observed (Fig. 7a). Figure 7b shows that the major part of carbon deposited on Ni4MgZr is amorphous like on NiZr, but very short filaments can be observed as well. The presence of carbon filaments increased progressively for Ni20MgZr and Ni40MgZr, with the filaments also becoming longer. It seems that the addition of MgO affected the carbon morphology, leading to more and longer filaments. Finally, in NiMg, only long filaments were observed.

#### TGA

**Fig. 8:**
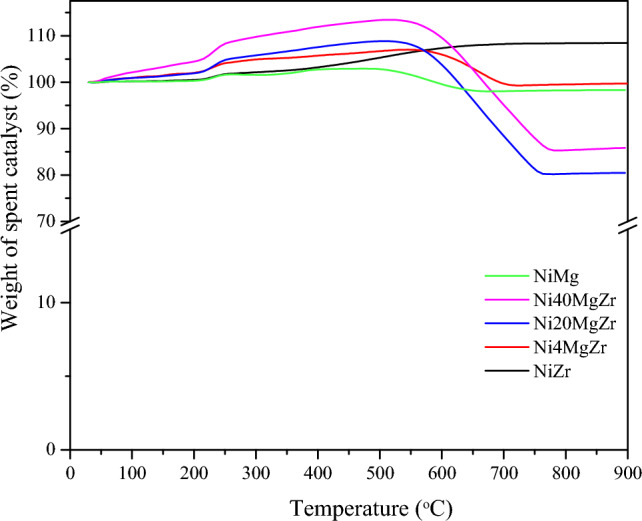
Results of thermogravimetric analysis (TGA) in air of used catalysts after 5 h of POM

Figure 8 shows the TGA profiles of catalysts used for 5 h of POM. The initial weight increase of used catalysts is related to the oxidation of Ni^0^ present after POM, with the exception of NiMg which does not contain Ni^0^. The following weight loss upon heating in air is then related to the gasification of carbon deposited on the catalyst (Fig. 8). Every used catalysts showed a pronounced weight loss between 500 and 750 °C, indicating that the coke formed was mainly graphitic carbon, typical of carbon with hierarchical structures (such as carbon nanotubes) [54].

According to the TGA profiles, the carbon-related weight loss of the used catalysts were: NiZr (not determined, shows an increase due to oxidation), Ni4MgZr (6%), Ni20MgZr (28%), Ni40MgZr (28%), and NiMg (5%). For NiMg, the carbon seems quite stable, as gasification was low. Roughly, the carbon-related weight loss follows the same trend as the carbon deposition rates in Table 5 (Ni20MgZr≈Ni40MgZr>>Ni4MgZr), corroborating that Ni4MgZr is the best catalyst for limiting carbon deposition during POM.

## Conclusions

Pre- and post-reaction analysis of various Ni-based catalysts, including different MgO–ZrO_2_ supports, and complemented by in situ XRD during H_2_ activation and POM reaction, yielded the following conclusions.

- In catalysts of NiO/MgO/ZrO_2_, synthesized by a one-step polymerization method and calcined at 750 °C, two solid-solutions were formed upon increasing MgO content: MgO–ZrO_2_ and NiO–MgO. Their presence was confirmed and characterized by XRD and XPS.
- Catalysts were pretreated by H_2_ reduction at 750 °C, which was monitored by H_2_-TPR and in situ XRD. With higher MgO content, the onset temperature of NiO reduction increased, but NiO persisted for NiO–MgO.
- In the partial oxidation of (bio)methane to syngas, Ni (30 wt%) supported on various MgO–ZrO_2_ solid solutions showed high methane conversion (~ 95%), good selectivity to H_2_ and CO (ratio of 2.2) and low carbon deposition rates, overall outperforming Ni/ZrO_2_.
- Ni4MgZr (4 mol% MgO) turned out to be the best catalyst. In situ XRD during POM revealed metallic Ni nanoparticles (average crystallite size of 31 nm), supported on MgO-ZrO_2_ solid solution, with small amounts of NiO–MgO being present as well.
- The amount of MgO in the Ni/MgO–ZrO_2_ catalysts also influenced the morphology of the carbon deposits. The more MgO, the more and longer carbon filaments were formed. The best catalyst (Ni4MgZr) predominantly produced amorphous carbon, with few short filaments.
- Although NiO-MgO also exhibited high conversion, it also suffered from rather high carbon deposition producing termally stable filaments.

## Data Availability

The data that support the findings of this study are available from the corresponding author upon reasonable request.
